# Optimization of magnetic flux density for fast MREIT conductivity imaging using multi-echo interleaved partial fourier acquisitions

**DOI:** 10.1186/1475-925X-12-82

**Published:** 2013-08-27

**Authors:** Munish Chauhan, Woo Chul Jeong, Hyung Joong Kim, Oh In Kwon, Eung Je Woo

**Affiliations:** 1 Department of Biomedical Engineering, Kyung Hee University, Yongin, Korea; 2 Department of Mathematics, Konkuk University, Seoul, Korea

**Keywords:** MREIT, MRI, Interleaved partial fourier acquisition, Magnetic flux density, Current density

## Abstract

**Background:**

Magnetic resonance electrical impedance tomography (MREIT) has been introduced as a non-invasive method for visualizing the internal conductivity and/or current density of an electrically conductive object by externally injected currents. The injected current through a pair of surface electrodes induces a magnetic flux density distribution inside the imaging object, which results in additional magnetic flux density. To measure the magnetic flux density signal in MREIT, the phase difference approach in an interleaved encoding scheme cancels out the systematic artifacts accumulated in phase signals and also reduces the random noise effect by doubling the measured magnetic flux density signal. For practical applications of *in vivo* MREIT, it is essential to reduce the scan duration maintaining spatial-resolution and sufficient contrast. In this paper, we optimize the magnetic flux density by using a fast gradient multi-echo MR pulse sequence. To recover the one component of magnetic flux density *B*_*z*_, we use a coupled partial Fourier acquisitions in the interleaved sense.

**Methods:**

To prove the proposed algorithm, we performed numerical simulations using a two-dimensional finite-element model. For a real experiment, we designed a phantom filled with a calibrated saline solution and located a rubber balloon inside the phantom. The rubber balloon was inflated by injecting the same saline solution during the MREIT imaging. We used the multi-echo fast low angle shot (FLASH) MR pulse sequence for MRI scan, which allows the reduction of measuring time without a substantial loss in image quality.

**Results:**

Under the assumption of *a priori* phase artifact map from a reference scan, we rigorously investigated the convergence ratio of the proposed method, which was closely related with the number of measured phase encode set and the frequency range of the background field inhomogeneity. In the phantom experiment with a partial Fourier acquisition, the total scan time was less than 6 seconds to measure the magnetic flux density *B*_*z*_ data with 128×128 spacial matrix size, where it required 10.24 seconds to fill the complete *k*-space region.

**Conclusion:**

Numerical simulation and experimental results demonstrated that the proposed method reduces the scanning time and provides the recovered *B*_*z*_ data comparable to what we obtained by measuring complete *k*-space data.

## Background

Magnetic resonance electrical impedance tomography (MREIT) utilizes a magnetic resonance imaging (MRI) scanner to measure magnetic flux density *B*_*z*_ data inside an imaging object induced by the externally injected current. The internal current density distribution has been studied in magnetic resonance current density imaging (MRCDI) by measuring the whole magnetic flux density data **B**=(*B*_*x*_,*B*_*y*_,*B*_*z*_) [1,2]. Combining MRCDI and electrical impedance tomography (EIT) technique, MREIT provides the cross-sectional conductivity images of the object with high spatial resolution [3-10]. Since an MRI scanner measures only one component *B*_*z*_ of **B** without rotating the imaging object, most MREIT algorithms assumed that the internal conductivity is isotropic and focused on visualizing its distribution by using one component of the magnetic flux density data *B*_*z*_ of **B**[11-18].

Recent MREIT imaging techniques have been developed with respect to both the capacity of measurement techniques and the numerical reconstruction algorithms. Experimental results from *in vivo* animal and human have been reported [19,20] in MREIT. As an innovation of current MREIT, a fast MREIT imaging technique referring to the continuous monitoring of objects includes various wide application areas [21]. Recently, one of challenging problem in MREIT is to implement a new imaging technique with a very short acquisition time for the imaging of neural activities of brain related to the conductivity change.

Since current MREIT experiments suffer from poor SNR of the measured *B*_*z*_ data under the typical data acquisition durations and a small amount of injected current, it is important to reduce the scan time, while maintaining the spatial-resolution and sufficient contrast, for practical implementations of *in vivo* MREIT. Recently, to reduce the scan time in MREIT, Hamamura *et al*[22] reconstructed the interior conductivity using a single-shot spin-echo echo planar imaging (SS-SEPI) pulse sequence and Muftuler *et al*[23] used a SENSE-accelerated imaging technique to acquire phase signal by the injected current. One of basic approaches for maintaining the spatial resolution is to reduce the number of phase encoding steps because each phase encoding step requires a certain amount of time for execution. Since the MREIT techniques use an interleaved phase encoding acquisition scheme to double the *B*_*z*_ signal, Park *et al*[24] reconstructed the phase signal *B*_*z*_ by filling the skipped *k*-space region using the interleaved measurement property.

To obtain the static conductivity image in MREIT [19,20], a spin-echo based MREIT pulse sequence has been predominantly used to reduce the background artifact and to increase the imaging quality. In real situations, it is difficult to employ the fast conventional MR pulse sequences because the noise standard deviation of *B*_*z*_ is inversely proportional to the width of injection current and the intensity of MR magnitude, simultaneously. An MREIT pulse sequence should be devised to enhance changes in MR phase images for given current amplitudes.

In this paper, we used the multi-echo fast low angle shot (FLASH) MR pulse sequence which allows the reduction of imaging time without any substantial loss in image quality. In addition, the multi-echo FLASH sequence maximizes the width of injection current extending the duration of injection current until the end of a readout gradient in MREIT. To reconstruct the internal conductivity distribution, most algorithms require at least two independent injection currents in an interleaved sense, which require relatively a long scanning duration [7,11]. To reduce the scanning time, we adopt a partial phase encoding acquisition scheme using the multi-echo FLASH MREIT pulse sequence and rigorously investigate the relationship between the convergence ratio of the algorithm and the background field inhomogeneity [24]. We consider the discrete *ℓ*^2^-norm to evaluate the convergence ratio.

To show the feasibility of the proposed algorithm, we performed numerical simulations and compared the performance to the simulated true *B*_*z*_ data. We designed a cylindrical acrylic phantom filled with a calibrated saline solution and located a rubber balloon inside the phantom. The rubber balloon was inflated by injecting the same saline solution during the scan. The phantom was designed to provide a homogeneous magnitude image, but distinguishable signals of measured *B*_*z*_ between the inside and outside the balloon. The phantom experiment demonstrated that the proposed method reduces the scanning time and recovers the reasonable resolution of *B*_*z*_, which is comparable to the recovered *B*_*z*_ using the complete *k*-space data.

## Method

### ***k***-space signal and ***B***_***z***_ data

In a conventional spin echo MREIT pulse sequence, both positive and negative currents of the same amplitude and duration are injected with reverse polarity. These injection currents with the pulse width of *T*_*c*_ accumulate extra phases. Corresponding *k*-space MR signals can be described as

(1) $$S^{\pm }(k_{x},k_{y})=\int_{\Omega }\rho (x,y)e^{\mathrm{i\delta}(x,y)}e^{\pm \mathrm{i\gamma}B_{z}(x,y)T_{c}}e^{i2\pi (k_{x}x+k_{y}y)}\text{dxdy}$$

where *ρ* is the *T*_2_ weighted spin density, *δ* is any systematic phase artifact, and *Ω* is a field-of-view (FOV). Here, the superscript of *S*^±^(*k*_*x*_,*k*_*y*_) denotes a brief notation for *S*^+^(*k*_*x*_,*k*_*y*_) and *S*^−^(*k*_*x*_,*k*_*y*_). For the standard coverage of *k*-space, we set

(2) $$\left\{\begin{array}{l} \;k_{x}=\frac{\gamma }{2\pi }G_{x}(\mathrm{n\Delta t}-T_{E})\;\text{for}\;n=-N_{x}/2,\cdots \;,N_{x}/2-1\\ \;k_{y}=\frac{\gamma }{2\pi }\mathrm{m\Delta}G_{y}T_{\text{pe}}\;\text{for}\;m=-N_{y}/2,\cdots \;,N_{y}/2-1 \end{array}\right)$$

where *γ*=26.75×10^7^rad/T·s is the gyromagnetic ratio of hydrogen, *Δt* is the time between samplings, *G*_*x*_ is the frequency encoding gradient strength, *T*_*E*_ is the echo time, *ΔG*_*y*_ is the phase encoding step, and *T*_*pe*_ is the phase encoding time. The induced magnetic flux density ±*B*_*z*_ is generated by the positive and negative injection currents *I*^±^. Applying the inverse Fourier transform to the measured *k*-space data sets in (1), we can compute the magnetic flux density *B*_*z*_ as

(3) $$B_{z}(r)=\frac{1}{2\gamma T_{c}}\tan^{-1}\left(\frac{\alpha (r)}{\beta (r)}\right)$$

where *α* and *β* are the imaginary and real part of $\left(\rho e^{\mathrm{i\delta}}e^{\mathrm{i\gamma}B_{z}T_{c}}\right)/\left(\rho e^{\mathrm{i\delta}}e^{-\mathrm{i\gamma}B_{z}T_{c}}\right)$, respectively [2].

The current MREIT method is based on the electromagnetic information embedded in the measured *B*_*z*_ data in order to visualize the conductivity(or current density) based on Biot-Savart law:

(4) $$B_{z}(r)=\frac{\mu_{0}}{4\pi }\int_{\Omega }\frac{\left(y-y^{\prime })J_{x}(r^{\prime })-(x-x^{\prime })J_{y}(r^{\prime }\right)}{|r-r^{\prime }{|}^{3}}dr^{\prime },\;r=(x,y,z),\;r^{\prime }=(x^{\prime },y^{\prime },z^{\prime })$$

where *μ*_0_=4*π*10^−7^Tm/A is the magnetic permeability of the free space. The current density **J** in *Ω* is given for the isotropic conductivity *σ* in *Ω*

(5) J(r)=−σ∇u(r)

and satisfies the following elliptic equation

(6) $$\begin{array}{l} \nabla \cdot \left(\sigma \nabla u\right)=0\;\text{in}\;\Omega \\ -\sigma \nabla u\cdot \nu =g\;\text{on}\;\partial \Omega \;\text{and}\;\int_{\partial \Omega }\;u\;\text{ds}=0 \end{array}$$

where *ν* is the outward unit normal vector on *∂Ω* and *g* is an applied current density on the surface.

### Recovery of complex $T_{2}^{*}$ weighted spin density using interleaved partial Fourier acquisition

To simplify, we develop a theory using a conventional cartesian *k*-space that has three zones within the *k*_*y*_(phase-encode) domain; the central region ($P_{0}$), the positive region ($P^{+}$) and the negative region ($P^{-}$):

(7) $$\left\{\begin{array}{l} \;P^{0}\;=\{(k_{x},k_{y})\;\left|\;k_{y}=\frac{\gamma }{2\pi }\mathrm{m\Delta}G_{y}T_{\text{pe}},\;-N_{c}\leq m\leq N_{c}\right)\}\\ \;P^{+}=\{(k_{x},k_{y})\;\left|\;k_{y}=\frac{\gamma }{2\pi }\mathrm{m\Delta}G_{y}T_{\text{pe}},\;m>N_{c}\right)\}\\ \;P^{-}=\{(k_{x},k_{y})\;\left|\;k_{y}=\frac{\gamma }{2\pi }\mathrm{m\Delta}G_{y}T_{\text{pe}},\;m<-N_{c}\right)\} \end{array}\right)$$

Here, *N*_*c*_ denotes the number of partial Fourier over-sampling phase-encodes. The interleaved *k*-space data $S_{p}^{+}(k_{x},k_{y})$ and $S_{p}^{-}(k_{x},k_{y})$ as partially acquired for the phase $k_{y}=\frac{\gamma }{2\pi }\mathrm{m\Delta}G_{y}T_{\text{pe}}$ can be expressed

(8) $$\begin{array}{ccc} S_{p}^{\pm }(k_{x},k_{y}) & = & \left\{\;,\begin{array}{ll} S^{\pm }(k_{x},k_{y}), & (k_{x},k_{y})\in P^{+}\cup P^{0}\\ 0, & (k_{x},k_{y})\in P^{-} \end{array}\right) \end{array}$$

where $S_{p}^{\pm }$ represents $S_{p}^{+}$ and $S_{p}^{-}$ simultaneously. We propose an algorithm to determine the $T_{2}^{*}$ weighted spin density *ρ* :

(9) $$\rho^{\pm }(r):=\rho (r)e^{\mathrm{i\delta}(r)}e^{\pm \mathrm{i\gamma}B_{z}(r)T_{c}}=FT^{-1}(S^{\pm }(k_{x},k_{y}))(r)$$

using the partially scanned *k*-space data $S_{p}^{\pm }$.

The systematic phase artifact *e*^*iδ*(*x*,*y*)^, unavoidable artifacts due to the main field inhomogeneity and the mismatch between the center of data acquisition interval and echo formation, arises from a low frequency field, which mainly belongs to the central region $P^{0}$. Including a small perturbed phase artifact *e*^*iδ*(*x*,*y*)^, we start with the initial guess $S_{p}^{\pm }$ and design an alternating procedure by updating the skipped *k*-space regions.

The recovered $\rho_{p}^{\pm }=FT^{-1}(S_{p}^{\pm })$ can be formally expressed by the equation

(10) $$\begin{array}{l} \rho_{p}^{\pm }(r)=FT^{-1}(S^{\pm }(k_{x},k_{y}))(r)+FT_{P^{-}}^{-1}(S_{p}^{\pm }(k_{x},k_{y}))(r)-FT_{P^{-}}^{-1}(S^{\pm }(k_{x},k_{y}))(r)\\ \;=\rho^{\pm }(r)+E_{p}^{\pm }(r) \end{array}$$

where $FT_{P^{-}}^{-1}(S_{p}^{\pm }(k_{x},k_{y}))$ denotes the inverse Fourier transform by zero-filling the *k*-space except in the region $P^{-}$ and the remainder term $E_{p}^{\pm }$ is

(11) $$E_{p}^{\pm }(r):=FT_{P^{-}}^{-1}\left(S_{p}^{\pm }(k_{x},k_{y})-S^{\pm }(k_{x},k_{y})\right)(r).$$

Let us define a support region $D_{\delta }$ for a low spatially varying magnetic field due to background field inhomogeneities:

(12) $$D_{\delta }:=\{(k_{x},k_{y})\;|\;\text{FT}(e^{-\mathrm{i\delta}})(k_{x},k_{y})=0,\;k_{y}=\frac{\gamma }{2\pi }\mathrm{m\Delta}G_{y}T_{\text{pe}},\;-N_{\delta }\leq m\leq N_{\delta }\}$$

**Observation 1.** If $D_{\delta }\subset P^{0}$, $\text{FT}(\rho_{p}^{\mp }e^{-2\mathrm{i\delta}})(k_{x},k_{y})=\text{FT}(\rho^{\mp }e^{-2\mathrm{i\delta}})(k_{x},k_{y})$ for $(k_{x},k_{y})\in P^{+}$.

The proof of observation 1 is provided in the Appendix A. By using the observation 1, we fill the skipped *k*-space regions in $S_{p}^{\pm }$

(13) $$\begin{array}{lll} S_{u}^{\pm }(k_{x},k_{y}) & = & \left\{\begin{array}{ll} \;S^{\pm }(k_{x},k_{y}),\;\; & (k_{x},k_{y})\in P^{+}\cup P^{0}\\ \;\bar{\text{FT}(\rho_{p}^{\mp }e^{-2\mathrm{i\delta}})}(-k_{x},-k_{y}), & (k_{x},k_{y})\in P^{-} \end{array}\right) \end{array}$$

**Observation 2.** If $D_{\delta }\subset P^{0}$, the *k*-space $S_{u}^{\pm }$ in recovers the *T*_2_ (or $T_{2}^{*}$) weighted spin density *ρ*^±^ without loss of information.

$$FT^{-1}(S_{u}^{\pm })=\rho^{\pm }=\rho e^{\mathrm{i\delta}}e^{\pm \mathrm{i\gamma}B_{z}(x,y)T_{c}}$$

The proof of observation 2 is provided in the Appendix 2. The observation 2 shows that the skipped region in the measured *k*-space *S*^+^ can be recovered by using the interleaved acquired *S*^−^ and the estimated background sensitivity map.

### Convergence characteristics

When the common measured $P^{0}$ does not cover the support region $D_{\delta }$, *N*_*δ*_>*N*_*c*_, for the phase-encode $(k_{x},k_{y})\in P^{+}$, the Fourier transform of $\rho_{p}^{-}e^{-2\mathrm{i\delta}}$ can be written by following the observation 1:

(14) $$\;\text{FT}(\rho_{p}^{-}e^{-2\mathrm{i\delta}})(k_{x},k_{y})=\underset{(k_{x},k_{m})\in P^{+}\cup P^{0}}{\sum }\text{FT}(\rho^{-})(k_{x},k_{m})\text{FT}(e^{-2\mathrm{i\delta}})(k_{x},k_{y}-k_{m})$$

From the relation (14), for the phase-encode $(k_{x},k_{y})\in P^{+}$, we have

(15) $$\begin{array}{l} \text{FT}(\rho^{-}e^{-2\mathrm{i\delta}})(k_{x},k_{y})-\text{FT}(\rho_{p}^{-}e^{-2\mathrm{i\delta}})(k_{x},k_{y})\\ =\sum_{(k_{x},k_{m})\in P^{-}}\text{FT}(\rho^{-})(k_{x},k_{m})\text{FT}(e^{-2\mathrm{i\delta}})(k_{x},k_{y}-k_{m}) \end{array}$$

We set

(16) $${\left[\psi \right]}_{R}:=FT^{-1}\left(\text{FT}(\psi ){|}_{R}\right)$$

where R is a subregion of the *k*-space and $\text{FT}(\psi ){|}_{R}$ denotes the restriction of *FT*(*ψ*) to the region R. The discrete *ℓ*_2_-norm of ${\left[\psi \right]}_{R}$ is equivalent to that of $\text{FT}(\psi ){|}_{R}$, *i.e.*, $∥{\left[\psi \right]}_{R}{∥}_{\ell_{2}}=C∥\text{FT}(\psi ){|}_{R}{∥}_{\ell_{2}}$, where the constant *C* is independent to the function *ψ* and the region R.

When the skipped *k*-space region, $P^{-}$, are filled the previously updated as in (13), we have

(17) $$\begin{array}{l} ∥{\left(\rho^{-}-\rho_{n}^{-}\right)}_{\ell_{2}}=C∥\text{FT}(\rho^{-}-\rho_{n}^{-}){∥}_{\ell_{2}}\\ \;=C∥\text{FT}(\rho^{-}-\rho_{n}^{-}){|}_{P^{-}}{∥}_{\ell_{2}}\\ \;=C{∥\left(\bar{\text{FT}(\rho^{+}e^{-2\mathrm{i\delta}})}-\bar{\text{FT}(\rho_{n-1}^{+}e^{-2\mathrm{i\delta}})}\right){|}_{P^{-}}(-k_{x},-k_{y})∥}_{\ell_{2}}\\ \;=C{∥\left(\text{FT}(\rho^{+}e^{-2\mathrm{i\delta}})-\text{FT}(\rho_{n-1}^{+}e^{-2\mathrm{i\delta}})\right){|}_{P^{+}}(k_{x},k_{y})∥}_{\ell_{2}}\\ \;=C{∥\left(\text{FT}((\rho^{+}-\rho_{n-1}^{+})e^{-2\mathrm{i\delta}})\right){|}_{P^{+}}(k_{x},k_{y})∥}_{\ell_{2}} \end{array}$$

For the *k*-space region $(k_{x},k_{y})\in P^{+}$, we have the following identity

(18) $$\begin{array}{l} \text{FT}(\left(\rho^{+}-\rho_{n-1}^{+})e^{-2\mathrm{i\delta}})\right){|}_{P^{+}}(k_{x},k_{y})\\ =\sum_{(k_{x},k_{m})\in P^{-}}\text{FT}(\rho^{+}-\rho_{n-1}^{+})(k_{x},k_{m})\text{FT}(e^{-2\mathrm{i\delta}})(k_{x},k_{y}-k_{m})\\ \;+\Theta (\rho^{+}-\rho_{n-1}^{+},P^{0}\cup P^{+}) \end{array}$$

where

$$\Theta (\rho^{+}-\rho_{n-1}^{+},P^{0}\cup P^{+}):=\underset{(k_{x},k_{m})\in P^{0}\cup P^{+}}{\sum }\text{FT}(\rho^{+}-\rho_{n-1}^{+})(k_{x},k_{m})\text{FT}(e^{-2\mathrm{i\delta}})(k_{x},k_{y}-k_{m}).$$

Since the updated complex density $\text{FT}(\rho_{n}^{\pm }){|}_{P^{+}\cup P^{0}}=\text{FT}(\rho^{\pm }){|}_{P^{+}\cup P^{0}}$, the remainder term $\Theta (\rho^{+}-\rho_{n-1}^{+},P^{0}\cup P^{+})=0$. Thus, the discrete *ℓ*^2^-norm of the difference between the true and the iteratively updated $T_{2}^{*}$ weighted spin density can be estimated

(19) $$\begin{array}{l} \;∥\left(\text{FT}((\rho^{+}-\rho_{n-1}^{+})e^{-2\mathrm{i\delta}})\right){|}_{P^{+}}{∥}_{\ell_{2}}^{2}\\ \leq ∥\text{FT}((\rho^{+}-\rho_{n-1}^{+}){|}_{P^{-}}{∥}_{\ell_{2}}^{2}\sum_{k_{y}\in P^{+}}\left[\sum_{(k_{x},k_{m})\in P^{-}}|{\text{FT}(e^{-2\mathrm{i\delta}})(k_{x},k_{y}-k_{m})|}^{2}\right] \end{array}$$

Detailed estimates of *ℓ*^2^-norm calculation are presented in the Appendix C Estimation of ***ℓ***^***2***^-norm.

Define an estimator for the convergence of the proposed algorithm

(20) $$Z_{2\delta ,P^{\pm }}:=\underset{k_{y}\in P^{+}}{\sum }\left[\underset{(k_{x},k_{m})\in P^{-}}{\sum }{\left|\text{FT}(e^{-2\mathrm{i\delta}})(k_{x},k_{y}-k_{m})\right|}^{2}\right]$$

Using the same procedures of (17) and (19), we have

(21) $$\begin{array}{c} ∥\text{FT}((\rho^{+}-\rho_{n-1}^{+}){|}_{P^{-}}{∥}_{\ell_{2}}^{2}=∥\left(\text{FT}((\rho^{-}-\rho_{n-2}^{-})e^{-2\mathrm{i\delta}})\right){|}_{P^{+}}(k_{x},k_{y}){∥}_{\ell_{2}}\\ \;\leq Z_{2\delta ,P^{\pm }}∥\text{FT}((\rho^{-}-\rho_{n-2}^{-}){|}_{P^{-}}{∥}_{\ell_{2}}^{2} \end{array}$$

The relations (17), (19) and (21) show that the convergence of the proposed method depends on the estimator $Z_{2\delta ,P^{\pm }}$:

(22) $$∥(\rho^{-}-\rho_{n}^{-}){∥}_{\ell_{2}}\leq Z_{2\delta ,P^{\pm }}∥(\rho^{-}-\rho_{n-2}^{-}){∥}_{\ell_{2}}$$

### Optimization of ***B***_***z***_ using gradient multi-echo data

Since the noise standard deviation $s_{B_{z}}$ of the measured *B*_*z*_ is inversely proportional to injection current duration *T*_*c*_ and the SNR of MR magnitude image *Υ*_*M*_[25,26] as

(23) $$s_{B_{z}}(r)=\frac{1}{\sqrt{2}T_{c}\Upsilon_{M}(r)}$$

To reduce the noise level of *B*_*z*_ and the imaging time, we applied the proposed method to the gradient multi-echo pulse sequence as a fast MR imaging technique. Subsequently, by using the gradient multi-echo, it is possible to inject the current for a long duration to maximize the off-resonance phase.

When $T_{E_{1}}$ denotes the first echo time and *ΔT*_*E*_ is the echo spacing, the *m*-th echo time is $T_{E_{m}}=T_{E_{1}}+(m-1)\Delta T_{E},\;m=1,\cdots \;,N_{E}$, where *N*_*E*_ is the echo number. The phase artifact $\delta_{T_{E_{m}}}$ depends on the echo time $T_{E_{m}}$. Using *a priori* estimation of the phase artifact map $\delta_{T_{E_{m}}}$ from a reference scan, for a time varying functional MREIT technique, the measured *k*-space region including $P^{0}$ and $P^{+}$ can be determined by taking account of the imaging multi-echo times $T_{E_{m}}$, the echo number *N*_*E*_ and the repetition time *T*_*R*_.

The recovered multiple $T_{2}^{*}$-weighted complex densities $\rho_{T_{E_{m}}}^{\pm },\;m=1,\cdots \;,N_{E},$ using the proposed algorithm in each echo time $T_{E_{m}}$ can be optimized to generate a representative measured $B_{z}^{m}$ data

(24) $$B_{z}^{m}(r)=\frac{1}{2\gamma T_{c}}\overset{-1}{\tan}\left(\frac{\alpha^{m}(r)}{\beta^{m}(r)}\right)$$

where *α*^*m*^ and *β*^*m*^ are the imaginary and real parts of $\rho_{T_{E_{m}}}^{+}/\rho_{T_{E_{m}}}^{-}$, respectively. The recovered multiple $B_{z}^{m}$ data include pixel-by-pixel different noise level depending on the different imaging time $T_{E_{m}}$ and the width of injection current. The multiple measured $B_{z}^{m}$ data are optimally combined to reduce the noise level of *B*_*z*_[27]:

(25) $$B_{z}(r)=\overset{N_{E}}{\underset{m=1}{\sum }}\omega_{m}(r)B_{z}^{m}(r)$$

where the point-wise weighting factor *ω*_*m*_(**r**) is given as

(26) $$\omega_{m}(r)=\frac{1/{\left(s_{B_{z}^{m}}(r)\right)}^{2}}{\overset{N_{E}}{\underset{k=1}{\sum }}1/{\left(s_{B_{z}^{k}}(r)\right)}^{2}}$$

Now, we setup an algorithm to reconstruct the magnetic flux density *B*_*z*_ data using the partially measured *k*-space data $S_{p}^{\pm }$ and involving the following steps: 

1. Take an initial guess $\rho_{0}^{\pm }=FT^{-1}(S^{\pm }{|}_{P^{0}\cup P^{+}})$.

2. Transform the *n*-th updated $\rho_{n}^{\pm }e^{-2\mathrm{i\delta}}$ to *k*-space by taking the Fourier transform.

3. Update the measured *k*-space data $S_{n+1}^{\pm }$ by filling the skipped region in $S_{p}^{\pm }$ with the transformed $\text{FT}(\rho_{n}^{\pm }e^{-2\mathrm{i\delta}})$ data.

4. Update $\rho_{n+1}^{\pm }$ by taking the two-dimensional inverse Fourier transform.

5. Stop if $\frac{∥\rho_{n+1}^{\pm }-\rho_{n}^{\pm }{∥}_{\Omega }}{∥\rho_{n}^{\pm }{∥}_{\Omega }}\leq \varepsilon$ where *ε*>0 is a given tolerance and ∥·∥_*Ω*_ is a standard *L*^2^-norm in *Ω*. Otherwise, repeat the process.

6. Reconstruct $B_{z}^{m}$ using (24) for *m*=1,2,⋯,*N*_*E*_.

7. Determine a weighting factor map *ω*_*m*_, *m*=1,2,⋯,*N*_*E*_ using (26) and reconstruct an optimally weighted *B*_*z*_ in (25).

### Experimental setup

#### Numerical simulation setup

**Figure 1:**
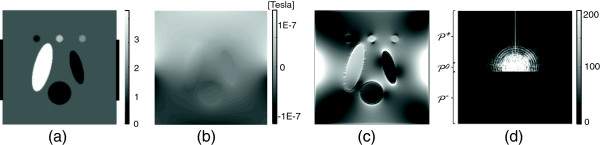
**Simulation setup.a)***T*_2_-weighted spin density, **b)** simulated magnetic flux density *B*_*z*_ image, **c)** intensity of |∇*B*_*z*_ image, **d)**magnitude image of the *k*-space data on the measured region.

To validate the proposed algorithm, we performed numerical simulations with the two-dimensional finite-element model of a object 20×20 cm^2^ with 256×256 rectangular elements and with the origin at its bottom-left, shown in Figure 1. We added the different complex field inhomogeneity artifacts to the simulated spin density image in Figure 1(a). The target magnetic flux density *B*_*z*_ in Figure 1(b) was generated by solving the elliptic equation (6) and by using the Biot-Savart law given by (4). Since the magnetic flux density *B*_*z*_ in Figure 1(b) is continuous and has no abrupt changes, we used |∇*B*_*z*_| image displayed in Figure 1(c) to enhance image for the magnetic flux density. Figure 1(d) shows a partially measured *k*-space data corresponding to *ρ*^+^.

The target conductivity distribution *σ* had different anomalies with different conductivity values and the amount of injection current was 10 mA. Set an applied current density *g* on the surface as

(27) $$g(x,y)=\left\{\begin{array}{l} \;10,\;\text{if}|y-5|\leq 2,\;\text{and}\;x=0\\ \;-10,\;\text{if}|y-5|\leq 2,\;\text{and}\;x=10\\ \;0,\;\text{otherwise.} \end{array}\right)$$

#### Phantom imaging experimental setup

**Figure 2:**
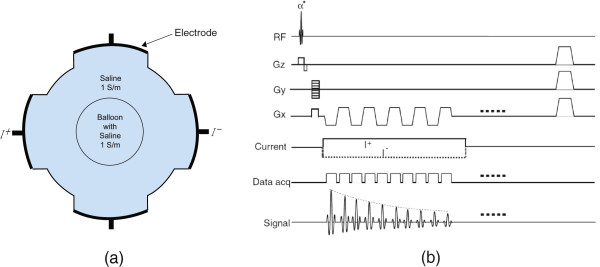
**Experimental setup.a)** saline phantom with balloon, **b)** diagram of the ICNE-multi-echo MR pulse sequence based on a gradient echo.

For the practical application of the proposed method as a fast MREIT imaging, we designed a cylindrical phantom filled with the saline solution of conductivity 1 S/m (shown in Figure 2(a)), including a rubber balloon for the visualization of isotropic conductivity excluding other artifacts by any concentration gradient in the phantom. The inside of balloon was filled with the same saline solution and the volume of balloon was controlled by injecting saline solution during the imaging experiment. After positioning the phantom inside the bore of 3T MR scanner (Achieva TX, Philips Medical Systems, Best, The Netherlands) with 8 channel RF coil, we collected *k*-space data using the gradient multi-echo injection current nonlinear encoding (ICNE) pulse sequence which was originated from FALSH (Figure 2(b)). To obtain the MR magnitude and magnetic flux density (*B*_*z*_) images, it extends throughout the duration of injection current until the end of a readout gradient [28]. Since the multi-echo ICNE pulse sequence was synchronized to the injection currents with alternating polarity, it enabled to maximize the width of the injection currents and minimize the noise standard deviation of the measured *B*_*z*_ data. The maximum amplitude of injection current was 5 mA and the total imaging time was 10.24 second to fill the *k*-space in the interleaved sense. Since the total imaging time was corresponding to the whole *k*-space scan, the actual imaging time would be reduced to 5.52 and 5.92 seconds for the number of partial region $N(P^{0}\cup P^{+})=69$ and 74, respectively. The imaging parameters were followings: slice thickness 5 mm, number of imaging slices one, repetition time *T*_*R*_=40 ms, echo spacing *ΔT*_*E*_=6 ms, flip angle 40 degree, and multi-echo time $T_{E_{m}}=6+(m-1)\times 6$ ms for *N*_*E*_=4. The FOV was 160 ×160 mm^2^ with a matrix size of 128×128. The duration of current injection $T_{c_{m}}$ was almost same to the multi-echo time $T_{E_{m}}=6+(m-1)\times 6,\;m=1,2,3,4$, because the current was continuously injected until the end of the readout gradient.

**Figure 3:**
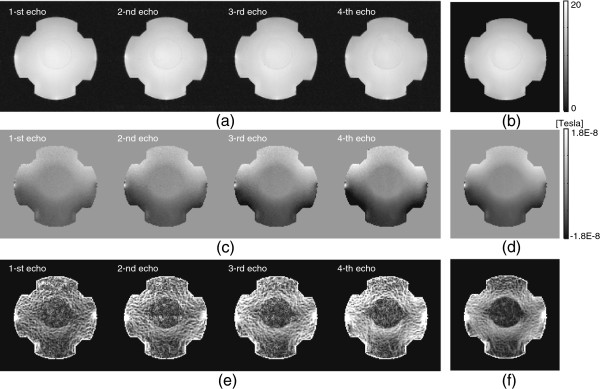
**Phantom imaging.a)**$T_{2}^{*}$-weighted magnitude images $|\rho_{T_{E_{m}}}^{+}|,\;m=1,\cdots \;,4$, **b)** averaged $T_{2}^{*}$ weighted magnitude image $\left|{\bar{\rho }}^{+}|:=\frac{1}{4}\overset{4}{\underset{m=1}{\sum }}|\rho_{T_{E_{m}}}^{+}\right|$, **c)** measured $B_{z}^{m}$ images at each echo *m*=1,⋯,4, **d)** averaged *B*_*z*_ image, $B_{z}:=\frac{1}{4}\overset{4}{\underset{m=1}{\sum }}B_{z}^{m}$, **e)**$\left|\nabla B_{z}^{m}\right|$ images at each echo *m*=1,⋯,4, **f)** averaged |∇*B*_*z*_| image, $\left|\nabla B_{z}|:=\frac{1}{4}\overset{4}{\underset{m=1}{\sum }}|\nabla B_{z}^{m}\right|$.

Figure 3(a) shows the multiply acquired magnitude images $|\rho_{T_{E_{m}}}^{+}|,\;m=1,\cdots \;,4$, where $\rho_{T_{E_{m}}}^{+}$ was the *m*-th measured $T_{2}^{*}$ weighted complex spin density, Figure 3(c) and (e) show the measured magnetic flux densities $B_{z}^{m}$ and the absolute of $\nabla B_{z}^{m}$ images at each echo *m*=1,⋯,4, respectively. The slope of $B_{z}^{m}$ reflecting the width of injected current linearly increased as the multi-echo time $T_{E_{m}}$ was increasing. Figure 3(b), (d), and (f) are the averaged images corresponding to Figure 3(a), (c), and (e), respectively.

## Results

### Simulation results

We compared the reconstructed magnetic flux density *B*_*z*_ using the complete *k*-space data to the *B*_*z*_ achieved using the partial *k*-space data. To evaluate the convergence characteristics of the proposed algorithm, we define the relative *ℓ*^2^-errors:

(28) $$E(\psi_{n}):=\frac{∥\psi -\psi_{n}∥}{∥\psi ∥}$$

where *ψ* and *ψ*^*n*^ are the recovered images with the complete *k*-space data and the *n*-th updated data using the partially measured *k*-space data, respectively, and ∥·∥ denotes the *ℓ*^2^-norm.

To investigate the estimator $Z_{2\delta ,P^{\pm }}$ for the convergence of the proposed iterative algorithm to fill the sipped *k*-space region $P^{-}$, we fixed the number of partially measured *k*-space region as $N(P^{0}\cup P^{+})=138$, *i.e.*, $N(P^{0})=20$ and $N(P^{+})=118$, and changed the frequency range of background field inhomogeneity. We generated several background field inhomogeneities changing the phase frequency range in *k*-space region by taking $N_{\delta }=5,10,20,30$ where the background field inhomogeneity *δ* satisfies *FT*(*e*^*iδ*^)(*k*_*x*_,*k*_*y*_)=0 for $|k_{y}|>N_{\delta }$.

**Figure 4:**
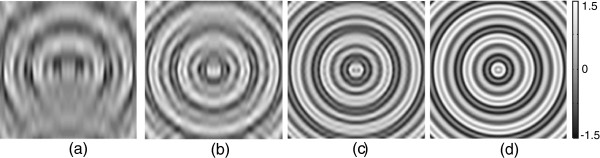
**Simulated background field inhomogeneity distributions.a**-**d)** real part images of *e*^*iδ*^ for $N_{\delta }=5,10,20,30$.

Figure 4(a)-(d) show the background field inhomogeneities used in the reconstruction procedure and Table 1 shows the estimated $Z_{2\delta ,P^{\pm }}$ in each background field inhomogeneity for $N_{\delta }=5,10,20,$ and 30, respectively.

**Table 1:** Calculated estimator$Z_{2\delta ,P^{\pm }}$ in (20) for$N_{\delta }=5,10,20,30$ and fixed measured***k***-space data $$N(P^{0}\cup P^{+})=138$$

	$N_{\delta }=5$	$N_{\delta }=10$	$N_{\delta }=20$	$N_{\delta }=30$
$Z_{2\delta ,P^{\pm }}$	0.0013	0.0749	0.4927	0.7738

**Figure 5:**
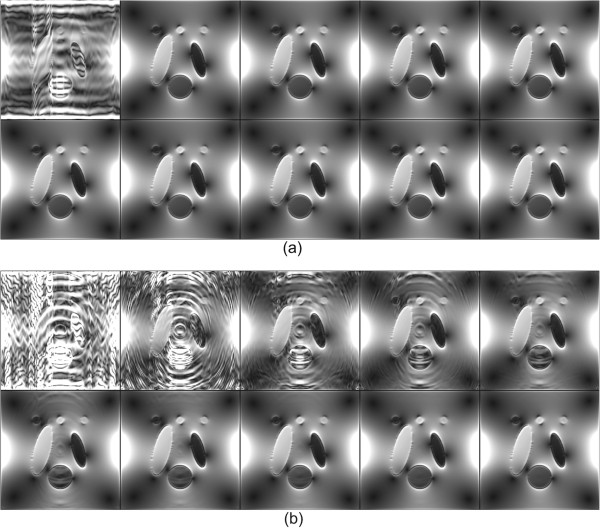
Reconstructed***|∇B***_*z*_| images using the fixed background field inhomogeneity with$N_{\delta }=5$ and$N_{\delta }=30$.**a)** reconstructed |∇*B*_*z*_| images using the fixed background field inhomogeneity with$N_{\delta }=5$. **b)** reconstructed |∇*B*_*z*_| images using the fixed background field inhomogeneity with$N_{\delta }=30$. From top-left to bottom-right, each image corresponds to the *j*-th iterative updated |∇*B*_*z*_|, *j*=0,1,⋯,9.

Figure 5(a) shows the reconstructed |∇*B*_*z*_| images using the fixed background field inhomogeneity with$N_{\delta }=5$. From the top-left to the bottom-right, each image was corresponding to the *j*-th iterative updated |∇*B*_*z*_|, *j*=0,1,⋯,10. The recovered *B*_*z*_ using the partially measured *k*-space data included a large amount of artifacts (the top-left image in Figure 5(a)). However, since the value of$Z_{2\delta ,P^{\pm }}$ was small, the first updated magnetic flux density almost recovered the true *B*_*z*_.

Figure 5(b) shows reconstructed |∇*B*_*z*_| images using the fixed background field inhomogeneity with$N_{\delta }=30$ corresponding to Figure 5(a). Since the value of$Z_{2\delta ,P^{\pm }}$ was 0.7738, the convergence ratio of *ρ*^±^ was relatively slow comparing to the field inhomogeneity with$N_{\delta }=5$. Table 2 shows the relative discrete *ℓ*^2^-errors of updated complex spin density for each iteration number and the convergence ratio of *ρ*^±^ was depending on the number of$N_{\delta }$.

**Table 2:** Relative*ℓ*^*2*^-errors of updated complex spin density for each iteration number(*♯*)

	***♯*0**	***♯*1**	***♯*2**	***♯*4**	***♯*6**	***♯*8**	***♯*10**
$E(\rho^{N_{\delta }=5})$	0.06291	0.00098	0.00044	0.00022	0.00021	0.00021	0.00021
$E(\rho^{N_{\delta }=10})$	0.08992	0.00282	0.00104	0.00039	0.00024	0.00020	0.00020
$E(\rho^{N_{\delta }=20})$	0.20801	0.03346	0.00706	0.00088	0.00043	0.00029	0.00023
$E(\rho^{N_{\delta }=30})$	0.22640	0.04623	0.01451	0.00309	0.00090	0.00041	0.00028

Table 3 shows the relative *ℓ*^2^-errors of reconstructed ∇*B*_*z*_ for each iteration number depending on the number of$N_{\delta }$. The decay rates of the relative *ℓ*^2^-error were very fast as the number of$N_{\delta }$ was small, but we needed relatively many iterations to approach the required accuracy as the number of$N_{\delta }$ was increase, even though the update procedure was rapidly computed by use of the fast Fourier transform.

**Table 3:** **Relative discrete*ℓ***^***2***^-errors of***∇B***_*z*_ for each iteration number(*♯*)

	***♯*0**	***♯*1**	***♯*2**	***♯*4**	***♯*6**	***♯*8**	***♯*10**
$E(\nabla B_{z}^{N_{\delta }=5})$	0.8755	0.0753	0.0462	0.0265	0.0214	0.0202	0.0199
$E(\nabla B_{z}^{N_{\delta }=10})$	1.0945	0.2189	0.0949	0.0552	0.0373	0.0288	0.0252
$E(\nabla B_{z}^{N_{\delta }=20})$	1.3132	0.6737	0.3039	0.1126	0.0730	0.0548	0.0435
$E(\nabla B_{z}^{N_{\delta }=30})$	1.3276	0.8300	0.4764	0.2242	0.1245	0.0823	0.0626

### Phantom experimental results

**Figure 6:**
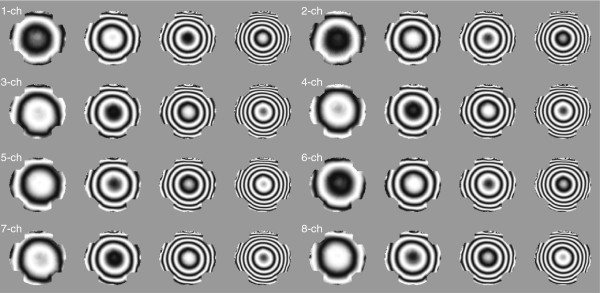
**Measured background field inhomogeneity distributions.** Real part of$e^{2i\delta_{\text{mn}}},\;n=1,\cdots \;,$*N*_*CH*_, *m*=1,⋯,*N*_*E*_, where *N*_*CH*_=8 and *N*_*E*_=4 denote the coil and echo numbers, respectively.

For the phantom experiment, we changed *N*_*c*_=5,⋯,10 for the set$P^{0}$ to investigate the convergence behavior with respect to a given background field inhomogeneity. Using the collected *k*-space data with 8 channel RF coil and the gradient multi-echo by alternating readout gradient, we measured the$T_{2}^{*}$ weighted complex densities$\rho_{m}^{\pm n},\;n=1,\cdots \;,N_{\text{CH}},\;m=1,\cdots \;,N_{E}$, where *N*_*CH*_=8 denotes the coil number and *N*_*E*_=4 is the echo number. Figure 6 shows the measured background field inhomogeneities by displaying the real part of$e^{2i\delta_{\text{mn}}}$ corresponding to the *n*-th coil and the *m*-th echo image. According to the increase of echo number, the accumulated background field inhomogeneity also increased.

**Figure 7:**
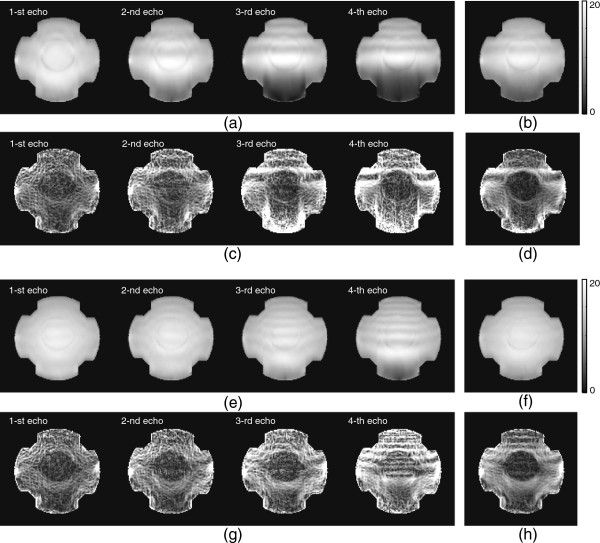
Measured$T_{2}^{*}$ weighted magnitude and magnetic flux density***B***_***z***_ images using$P^{0}\cup P^{+}$ with***N***_***c***_***=5* and*N***_***c***_***=10***.**a)** and **e)**$T_{2}^{*}$ weighted magnitude image at each echo time$T_{E_{m}},\;m=1,2,3,4$, with *N*_*c*_=5 and *N*_*c*_=10, respectively. **b)** and **f)** combined$T_{2}^{*}$ weighted magnitude image with *N*_*c*_=5 and *N*_*c*_=10, respectively. **c)** and **g)** recovered *B*_*z*_ image at each echo time$T_{E_{m}},\;m=1,2,3,4$ with *N*_*c*_=5 and *N*_*c*_=10, respectively. **d)** and **h)** weighted *B*_*z*_ image using multiple *B*_*z*_ image at each echo time with *N*_*c*_=5 and *N*_*c*_=10, respectively.

Figure 7(a) and (c) show the measured$T_{2}^{*}$ weighted magnitude and magnetic flux density *B*_*z*_ images at the time$T_{E_{m}},\;m=1,2,3,4$, using partially acquired *k*-space region$P^{0}\cup P^{+}$ with *N*_*c*_=5 by a transversally injected current. Although the amount of accumulated phase signal by the injected current increased as the echo time varied from$T_{E_{1}}$ to$T_{E_{4}}$, the magnitude image at the 4-th echo was more deteriorated comparing to the 1-st echo case. Figure 7(b) shows an averaged MR magnitude image at each echo time and Figure 7(d) is a weighted *B*_*z*_ image depending on the width of injected current using the phase signal in Figure 7(c). Figure 7(e)-(h) shows the measured magnitude and magnetic flux density *B*_*z*_ images corresponding to Figure 7(a)-(d) using partially acquired *k*-space region$P^{0}\cup P^{+}$ with *N*_*c*_=10.

Comparing to the measured images in Figure 7(a)-(d), in contrast to the recovery of low phase frequency information corresponding to$P^{0}$, the increased background field inhomogeneity caused relatively high frequency artifacts.

**Figure 8:**
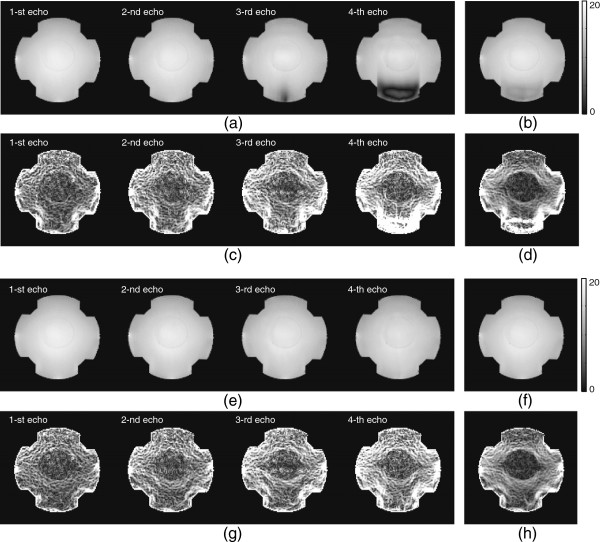
**Iteratively updated**$T_{2}^{*}$ weighted magnitude and magnetic flux density*B*_*z*_ images using$P^{0}\cup P^{+}$ with*N*_*c*_*=5* and*N*_*c*_*=10*.**a)** and **e)** recovered$T_{2}^{*}$ weighted magnitude image at each echo time$T_{E_{m}},\;m=1,2,3,4$, with *N*_*c*_=5 and *N*_*c*_=10, respectively. **b)** and **f)** combined$T_{2}^{*}$ weighted magnitude image using the recovered magnitude image at each echo time with *N*_*c*_=5 and *N*_*c*_=10, respectively. **c)** and **g)** recovered *B*_*z*_ image at each echo time$T_{E_{m}},\;m=1,2,3,4$,with *N*_*c*_=5 and *N*_*c*_=10, respectively. **d)** and **h)** weighted *B*_*z*_ image using multiple *B*_*z*_ image at each echo time with *N*_*c*_=5 and *N*_*c*_=10, respectively.

Figure 8(a)-(d) shows iteratively updated$T_{2}^{*}$ weighted magnitude and magnetic flux density *B*_*z*_ images using$P^{0}\cup P^{+}$ with *N*_*c*_=5. We fixed the update iteration number as 20 for all experiments. When we fixed *N*_*c*_=5, the 1-st and 2-nd recovered$T_{2}^{*}$ weighted complex densities in Figure 8(a) and (c) were relatively close to the recovered ones using the complete *k*-space data. However, as the phase artifact increased, the 3-rd and 4-th recovered$T_{2}^{*}$ weighted complex densities were deficient in reflecting full information of *B*_*z*_ signal. Especially, the 4-th updated magnetic flux density *B*_*z*_ image shows some defective region due to the insufficient recovery of$T_{2}^{*}$ weighted complex density.

Figure 8(e)-(f) shows iteratively updated$T_{2}^{*}$ weighted magnitude and magnetic flux density *B*_*z*_ images corresponding to Figure 8(a)-(d) using$P^{0}\cup P^{+}$ with *N*_*c*_=10. When we used *N*_*c*_=10, the updated$T_{2}^{*}$ weighted complex densities almost recovered the magnetic flux density *B*_*z*_ data comparing to those using the complete *k*-space data.

## Discussion

We used the gradient multi-echo MREIT pulse sequence to reduce the imaging time and to maximize injection current duration. Since the MREIT techniques utilize accumulated phase signal by the injected current, it requires enough repetition time *T*_*R*_ to accumulate the phase signal. In this sense, the gradient multi-echo MREIT pulse sequence seems practical approach for the improvement of *B*_*z*_ quality as well as reducing the imaging time. In this paper, we used a partially acquired *k*-space data in the phantom experiment by filling the *k*-space as much as 74 line by line, results in 5.92 second to image the resolution of 128×128. Experimental results show that the proposed interleaved partial Fourier strategy for MREIT has a potential to reduce scan times and maintain the information of *Bz* data comparable to what is obtained with complete *k*-space data.

The convergence ratio of the iteratively updated phase signal heavily depends on the frequency of the background filed inhomogeneity and the number of half-Fourier over-sampling phase-encodes$P^{0}$. Instead of the gradient multi-echo, if we use the spin multi-echo pulse sequence, the proposed iterative algorithm would rapidly recover *T*_2_-weighted complex spin density due to a small amount of background field inhomogeneity. However, in spite of some advantages of the spin multi-echo MREIT pulse sequence, for a real-time MREIT imaging, MR pulse sequence should be carefully investigate by taking into account of the width of injection current, the scan duration and the low SNR of measured *B*_z_ signal.

In this paper, we assumed *a priori* background field inhomogeneity which is typically used in the sensitivity encoding (SENSE) as a fast MRI measurement technique. Since the MREIT techniques typically used interleaved acquisition by injecting alternative currents, it may be possible to extract background field inhomogeneity information under a low frequency range assumption and by cancelation of *B*_z_ information:

(29) $$\begin{array}{lll} \rho^{+}(x,y)\rho^{-}(x,y) & = & \rho^{2}(x,y)e^{2\mathrm{i\delta}(x,y)}\\ \frac{\rho^{+}(x,y)}{\rho^{-}(x,y)} & = & e^{2\mathrm{i\gamma}B_{z}(x,y)T_{c}} \end{array}$$

Several studies reported for the feasibility of MREIT to detect neural activities in the brain, directly [29,30]. Functional MREIT technique is suggested to image brain activity *via* conductivity change related to neural activity through the fast MREIT pulse sequence. Our future study will focus on applying the proposed method to produce functional conductivity images of animal and/or human brain to pursue rapidly changing conductivity associated with neural activities.

## Conclusion

In MREIT, the inherent challenges are to reduce the scan time and maintain current injection duration to make it feasible for the clinical applications. We developed an iterative method to optimize the measured magnetic flux density *B*_z_ using the multi-echo interleaved partial Fourier acquisitions for fast imaging in MREIT. The proposed method used a fast gradient multi-echo MR pulse sequence to reduce the scan time and to maximize the phase signal by injection current. Under the assumption of *a priori* background field inhomogeneity map, we rigorously investigated the convergence ratio of the proposed method using the discrete *ℓ*^2^-norm, which was closely related with the number of measured phase encode set and the frequency range of the background field inhomogeneity. To evaluate the proposed method, a specially designed conductivity phantom was used to provide a homogeneous magnitude, but it yielded distinguishable *B*_z_ signal inside and outside the anomaly. For the phantom experiment, total imaging time was 10.24 seconds to fill the complete *k*-space region in the interleaved sense and it was less than 6 seconds to fill the partial *k*-space region to implement the proposed method. The proposed interleaved partial Fourier strategy for the fast MREIT has a potential to reduce scan times and maintain the information of *B*_z_ data comparable to what is obtained with the complete *k*-space data.

## Appendix

## A Proof of Observation 1

For the phase-encode$(k_{x},k_{y})\in P^{+}$, the Fourier transform of$\rho_{p}^{-}e^{-2\mathrm{i\delta}}$ can be separated as

(30) $$\;\begin{array}{ll} \text{FT}(\rho_{p}^{-}e^{-2\mathrm{i\delta}})(k_{x},k_{y}) & =(\text{FT}(\rho_{p}^{-})*\text{FT}(e^{-2\mathrm{i\delta}}))(k_{x},k_{y})\\ & =\sum_{(k_{x},k_{m})\in P^{+}\cup P^{0}\cup P^{-}}\text{FT}(\rho_{p}^{-})(k_{x},k_{m})\text{FT}(e^{-2\mathrm{i\delta}})(k_{x},k_{y}-k_{m})\\ & =\sum_{(k_{x},k_{m})\in P^{+}\cup P^{0}}\text{FT}(\rho_{p}^{-})(k_{x},k_{m})\text{FT}(e^{-2\mathrm{i\delta}})(k_{x},k_{y}-k_{m})\\ & \;+\sum_{(k_{x},k_{m})\in P^{-}}\text{FT}(\rho_{p}^{-})(k_{x},k_{m})\text{FT}(e^{-2\mathrm{i\delta}})(k_{x},k_{y}-k_{m}) \end{array}$$

where ∗ denotes the convolution with respect to *k*_*y*_. Since the updated$S_{p}^{-}$ data conserve the measured data in$P^{+}\cup P^{0}$,$\text{FT}(\rho_{p}^{-})(k_{x},k_{y})=\text{FT}(\rho^{-})(k_{x},k_{y})$ for$(k_{x},k_{y})\in P^{+}\cup P^{0}$. Thus, we have

(31) $$\;\begin{array}{ll} \text{FT}(\rho_{p}^{-}e^{-2\mathrm{i\delta}})(k_{x},k_{y})= & \sum_{(k_{x},k_{m})\in P^{+}\cup P^{0}}\text{FT}(\rho^{-})(k_{x},k_{m})\text{FT}(e^{-2\mathrm{i\delta}})(k_{x},k_{y}-k_{m})\\ & \;+\sum_{(k_{x},k_{m})\in P^{-}}\text{FT}(\rho_{p}^{-})(k_{x},k_{m})\text{FT}(e^{-2\mathrm{i\delta}})(k_{x},k_{y}-k_{m})\\ & =\sum_{(k_{x},k_{m})\in P^{+}\cup P^{0}}\text{FT}(\rho^{-})(k_{x},k_{m})\text{FT}(e^{-2\mathrm{i\delta}})(k_{x},k_{y}-k_{m}) \end{array}$$

Since the central phase-encode set$P^{0}$ includes all phase frequencies of the systematic phase artifact *e*^−*iδ*^, the range of the phase frequency *k*_*y*_−*k*_*m*_ for$(k_{x},k_{y})\in P^{+}$ and$(k_{x},k_{m})\in P^{-}$ is over 2*N*_δ_. This means that *FT*(*e*^−2*i*δ^)(*k*_*x*_,*k*_*y*_−*k*_*m*_)=0. Thus, we have$\text{FT}(\rho_{p}^{-}e^{-2\mathrm{i\delta}})(k_{x},k_{y})=\text{FT}(\rho^{-}e^{-2\mathrm{i\delta}})(k_{x},k_{y})$ for$(k_{x},k_{y})\in P^{+}$. The case for$\rho_{p}^{+}e^{-2\mathrm{i\delta}}$ is similar.

## B Proof of Observation 2

Since$\text{FT}(\rho_{p}^{\mp }e^{-2\mathrm{i\delta}})(k_{x},k_{y})=\text{FT}(\rho^{\mp }e^{-2\mathrm{i\delta}})(k_{x},k_{y})$ for$(k_{x},k_{y})\in P^{+}$ due to the observation 1, we have

(32) $$\text{FT}(\rho^{+}e^{-2\mathrm{i\delta}})(k_{x},k_{y})=\text{FT}(\rho e^{-\mathrm{i\delta}}e^{\mathrm{i\gamma}B_{z}T_{c}})(k_{x},k_{y})\;\text{for}\;(k_{x},k_{y})\in P^{+}$$

From the relation (32), by taking the complex conjugate, we recover the skipped *k*-space region$P^{-}$

(33) $$\begin{array}{l} \text{FT}(\rho^{-})(k_{x},k_{y})=\text{FT}(\rho e^{\mathrm{i\delta}}e^{-\mathrm{i\gamma}B_{z}T_{c}})(k_{x},k_{y})\\ \;=\bar{\text{FT}(\rho e^{-\mathrm{i\delta}}e^{\mathrm{i\gamma}B_{z}T_{c}})}(-k_{x},-k_{y})\\ \;=\bar{\text{FT}(\rho_{p}^{+}e^{-2\mathrm{i\delta}})}(-k_{x},-k_{y}) \end{array}$$

## C Estimation of **ℓ**^**2**^**-norm**

The discrete ℓ^2^-norm of the difference between the true and the iteratively updated$T_{2}^{*}$ weighted spin density can be estimated as following:

(34) $$\begin{array}{l} ∥\left(\text{FT}((\rho^{+}-\rho_{n-1}^{+})e^{-2\mathrm{i\delta}})\right){|}_{P^{+}}{∥}_{\ell_{2}}^{2}\\ =\sum_{k_{y}\in P^{+}}|\text{FT}\left((\rho^{+}-\rho_{n-1}^{+})e^{-2\mathrm{i\delta}}\right)(k_{x},k_{y}){|}^{2}\\ =\sum_{k_{y}\in P^{+}}{\left|\sum_{(k_{x},k_{m})\in P^{-}}\text{FT}(\rho^{+}-\rho_{n-1}^{+})(k_{x},k_{m})\text{FT}(e^{-2\mathrm{i\delta}})(k_{x},k_{y}-k_{m})\right|}^{2}\\ \leq \sum_{k_{y}\in P^{+}}{\left[\sum_{(k_{x},k_{m})\in P^{-}}|\text{FT}(\rho^{+}-\rho_{n-1}^{+})(k_{x},k_{m})\text{FT}(e^{-2\mathrm{i\delta}})(k_{x},k_{y}-k_{m})|\right]}^{2}\\ \leq \sum_{k_{y}\in P^{+}}\left[∥\text{FT}((\rho^{+}-\rho_{n-1}^{+}){|}_{P^{-}}{∥}_{\ell_{2}}^{2}\sum_{(k_{x},k_{m})\in P^{-}}|\text{FT}(e^{-2\mathrm{i\delta}})(k_{x},k_{y}-k_{m}){|}^{2}\right]\\ \leq ∥\text{FT}((\rho^{+}-\rho_{n-1}^{+}){|}_{P^{-}}{∥}_{\ell_{2}}^{2}\sum_{k_{y}\in P^{+}}\left[\sum_{(k_{x},k_{m})\in P^{-}}|\text{FT}(e^{-2\mathrm{i\delta}})(k_{x},k_{y}-k_{m}){|}^{2}\right] \end{array}$$

## Abbreviations

MRI: Magnetic resonance imaging; MREIT: Magnetic resonance electrical Impedance Tomography; MRCDI: Magnetic resonance current density imaging; EIT: Electrical impedance tomography; SNR: Signal-to-noise ratio; SS-SEPI: Single-shot Spin-echo Echo Planar Imaging; FOV: Field of view; ICNE: Injection current nonlinear encoding.

## Competing interests

The authors declare that they have no competing interests.

## Authors’ contributions

All authors were involved in the analysis of numerical and experimental data. All authors were involved in the preparing of the manuscript. All authors read and approved the final manuscript.
